# Colchicine-Induced Polyploidy in Leguminous Crops Enhances Morpho-Physiological Characteristics for Drought Stress Tolerance

**DOI:** 10.3390/life13101966

**Published:** 2023-09-26

**Authors:** Phetole Mangena, Pirtunia Nyadzani Mushadu

**Affiliations:** Department of Biodiversity, Faculty of Science and Agriculture, School of Molecular and Life Sciences, University of Limpopo, Private Bag X1106, Sovenga 0727, South Africa; nyadzanimushadu@gmail.com

**Keywords:** colchicine, drought stress, legumes, morphological traits, physiological traits, induced polyploidy

## Abstract

Legumes play a significant role in the alleviation of food insecurity, maintaining soil fertility, and achieving sustainable crop production under adverse environmental conditions. The increased demand in legume production contemplates that attention on the genetic improvement of these crops through various means such as genetic engineering and mutation breeding should take a centre stage in global agriculture. Therefore, this paper provides a succinct analysis of the currently available literature on morphological and physiological traits in polyploidised leguminous plants to counter the adverse effects of drought stress. The effects of colchicine on various morphological and physiological traits of polyploidised legumes compared to their diploid counterparts were examined. Numerous reports revealed variations in these traits, such as improved root and shoot growth, plant biomass, chloroplastidic content, protein, RNA, and DNA. The differences observed were also associated with the strong relationship between plant ploidy induction and colchicine application. Furthermore, the analysis indicated that polyploidisation remains dose-dependent and may be achievable within a shorter space of time as this antimitotic chemical interferes with chromosome separations in somatic plant cells. The efficiency of this process also depends on the advancement of treatment conditions (in vitro, in vivo, or ex vitro) and the successful regeneration of polyploidised plants for adaptation under drought stress conditions. As such, the improvement in metabolite profile and other essential growth characteristics serves as a clear indication that induced polyploidy needs to be further explored to confer resilience to environmental stress and improve crop yield under drought stress conditions in leguminous plants.

## 1. Introduction

The global demand for legumes as foods, feeds, excellent sources of bioactive health compounds, and raw materials for industrial manufacturing continues to grow immensely due to climate change and increasing human populations [1]. Generally, this rapid population upsurge, malnutrition, poverty, unemployment, and climate-change-driven biotic as well as abiotic stress factors dramatically raise the pressure on the food systems, especially agricultural legume production [2]. Nevertheless, the inclusion of improved leguminous crop varieties in cropping systems plays a significant role in alleviating the abovementioned challenges, particularly, global issues such as food insecurity, decreasing soil fertility, and developing climate-resilient cultivars that can be grown under adverse environmental conditions [3,4].

Plant polyploidisation using antimitotic chemicals such as colchicine serves as one of the techniques that are not yet fully explored for varietal improvements and the generation of basic genetic information of grain legumes, such as cowpea (*Vigna unguiculata* (L.) Walp.), faba bean (*Vicia faba* L.), and soybean (*Glycine max* (L.) Merrill.), for enhanced growth and resilience to abiotic stresses like drought, salinity, and waterlogging. Polyploidisation, induced in vitro, in vivo, or ex vitro, can cause desirable changes to the morphology, anatomy, physiology, and genomic makeup of crop species, as this technique serves as a viable alternative to genetic engineering technologies such as biolistic microprojectile bombardment, electroporation, CRISPR-Cas, and indirect *Agrobacterium tumefaciens*-mediated gene transfer methods [5,6]. Although biolistic delivery of exogenous DNA into host plant cells has been widely explored for the genetic improvement of soybeans and other leguminous and non-leguminous crops, its inconsistencies among bombarded samples for transient gene expression analysis often hinder the quantitative determination of putative transgenic plants [5]. 

**Figure 1:**
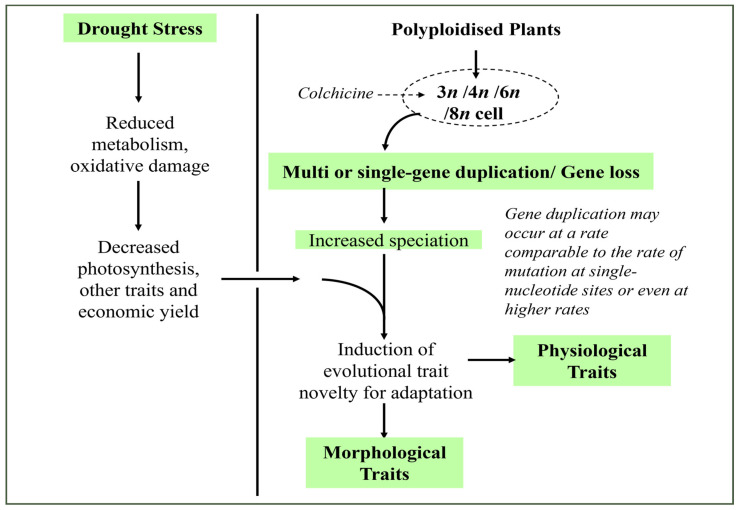
A model for the role of synthetic polyploidy in counteracting abiotic stress (drought) effects in plants. The single/multiple gene segment duplication may lead to changes in the genome with unique evolutionary traits such as increased morphological and physiological functions that are beneficial [9,10].

The utility of this approach has also been applied to determine the efficiency of multiple gRNAs for CRISPR-Cas9-mediated gene transfer, as also alluded to by Hadama et al. [7] and Lui et al. [8]. These modern genetic manipulation technologies remain constrained by the over-pronounced recalcitrance of crop species, high genotype dependency, and excessive costs of operations that render them inaccessible. However, various reports have highlighted that pretreatment of crop seeds with appropriate concentrations of colchicine and duration and immersion conditions may result in the development of morphologically, anatomically, and physiologically modified plants, especially in recalcitrant legumes such as soybean, chickpea (*Cicer arietinum*), pea (*Pisum sativum*), lentil (*Lens culinaris* Medikus), and tepary bean (*Phaseolus acutifolius*) (Figure 1). Although high dosages may be toxic and lethal to plant cells, the reports have also shown that colchicine still contributes to the positive regulation of germination, growth speed, and reproductive processes of treated crop plants even under drought stress conditions, as shown in Figure 1 [9,10,11]. 

Ploidy induction, as referred to by Yadav et al. [11], instantaneously leads to the formation of new genomic structures or speciation (Figure 1) wherein the individual offspring may become ecologically and epigenetically unique from their diploid counterparts. As would be desired by many plant breeders, this provides new lineages upon which further selection and breeding can be carried out for the development of newly improved varieties. However, colchicine-induced polyploidisation in leguminous plant species remains one of the topics scantly researched and reported. As a result of this, the present paper focuses on the progress, achievements, constraints, and perspectives of using colchicine-induced polyploidisation technology to improve the growth and yield of leguminous crop species under drought stress conditions. This paper advocates for the application of colchicine as a plant breeding chemical agent and provides important insights that could encourage the expanded cultivation of legumes in drought-affected agricultural fields.

## 2. Role of Drought Stress on Growth and Yield of Leguminous Crops

Drought serves as one of the most important abiotic constraints that inflict severe damaging effects on the growth and yield of leguminous crops. This stress has been reported as an inevitable factor that exists in various environments [12], negatively influencing crop production, biomass, and yield quality by altering morphological, physiological, and molecular responses in affected plants [13]. Table 1 shows a summary of some of the growth and developmental aspects of plants that are negatively impacted or increased (i.e., floral/pod abortions, generation of reactive oxygen species (ROS), proline content, and malonaldehyde content) because of the exposure of plants to drought stress. Many legumes and non-leguminous crops exhibit these high levels of sensitivity and susceptibility to drought stress more than any kind of abiotic constraint. In legumes, crop growth and yields are severely impacted by inadequate supply of water, which results in decreased carbon assimilation content and rates. This deadly impact and these reducing effects take place regardless of the stage of plant growth [14].

Generally, all crop plants that are cultivated under open environments remain at a high risk of passing through a brief or prolonged period of abiotic stress at any point during their life cycle. However, different crops exhibit varying sensitivity and susceptibility to drought stress and present dissimilar effects on the metabolic activities, growth, and development of individual genotypes. In addition to leaf photosynthetic capacity, shoot biomass, and seed weight, drought stress disrupted sucrose metabolism and the transport balance of photoassimilates in both leaves and seeds of soybeans [15]. Drought also decreased the yield of chickpea (*Cicer arietinum*) by about 45–50% through interference with seedlings and early vegetative growth stages. Under high temperatures, which also exacerbate water deficits, chickpea also suffered severe cytoplasmic water losses that concomitantly led to flower and fruit abortions, reduced seed sizes, loss of pollen viability and fertility that affected pod sets, and ultimately reduced the yield of this crop [16]. 

Apart from the effects mentioned above, drought can have massive destructive effects on the growth and development of commercially valuable leguminous crops such as lentils, faba bean, common bean (*Phaseolus vulgaris*), and cowpea, with higher magnitude impairments in the source and sink relations during the vegetative and reproductive growth stages of the plants [17,18,19,20]. In comparison, drought-stressed plants showed more reduced morphological traits, such as the leaf area, number of branches, stem diameter, and root length, than water-stress-free leguminous plants. Additionally, the activities of superoxide dismutase (SOD), peroxidase (POD), and catalase (CAT) together with malonaldehyde (MDA) content were also increased, as illustrated in Table 1 [15]. Drought stress also significantly decreases the accumulation of biochemical substances (Table 1) which inevitably impairs metabolism and photosynthetic activities, leading to plant mortality [21,22].

**Table 1:** Aspects of plant growth and development influenced by drought stress in both leguminous and non-leguminous crop species.

Growth	Reproductive	Physiological	Biochemical
Seed germination, plant height, number of branches, number of leaves, stem diameter, leaf area, root development, and nodulation	Flowering, flower abortion, fruiting, pod abortion, number of seeds per pod, pod size, seed size, 100-seed weight, and yield per hectare	Antioxidant, catalases, glutathione/ascorbate-glutathione, superoxide dismutase, inorganic ions (Na^+^, K^+^, N, Ca, Mg, Fe, Zn, Mn, and B), reactive oxygen species (ROS)	Abscisic acid (ABA), amino acids, carotenoids, DNA, RNA, proteins, proline content, reducing sugars, non-reducing sugars, proline, starch, chlorophyll a, chlorophyll b, malonaldehyde, phenols, and alkaloids.

Sources: Biochemical [23], growth [24,25], physiological [25,26], and reproductive parameters [15,27].

## 3. Impact of Polyploidisation on Alleviating the Effects of Drought Stress

Most leguminous crops are well adapted to tropical and subtropical environmental conditions. However, the magnitude of abiotic stresses that occur in these regions results in massive losses of yields, with drought being the main contributor to many previously recorded reductions in crop production [28]. As more incidences of drought continue to occur, circumventing the vulnerabilities associated with crop production under drought stress conditions remains a key research priority. In this regard, the expansion of germplasm through synthetic crop polyploidisation using mutagenic chemicals such as colchicine could represent a viable alternative for biotechnology-based genetic improvements. Colchicine is an alkaloid that is usually extracted from a Colchicaceae species, *Gloriosa superba* L. (Flame Lily), the only species in this genus, and order Liliales. This chemical is also present in the seeds of *Colchicum autumnale* L. and *Colchicum luteum* L. [29]. 

**Figure 2:**
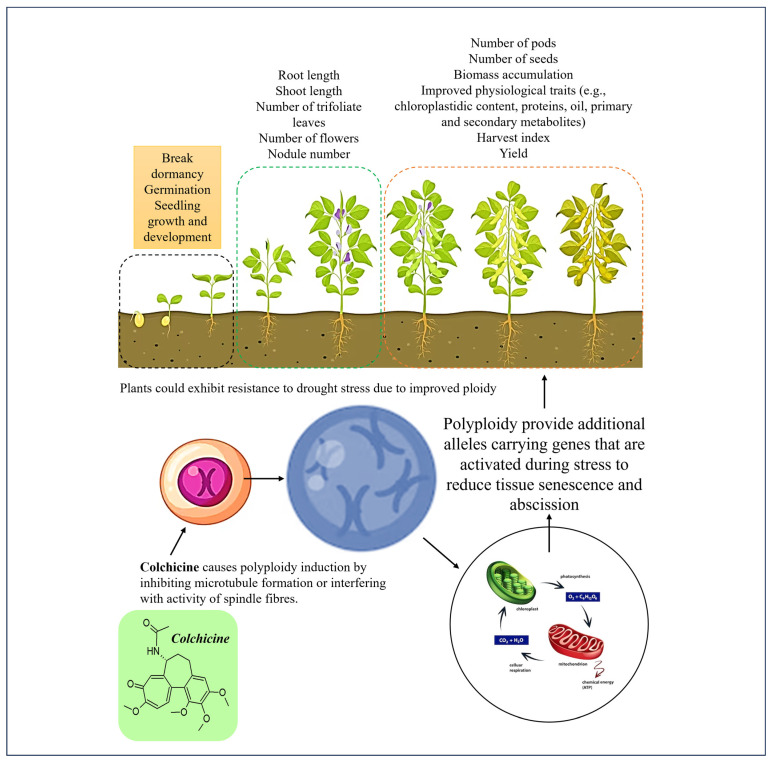
Demonstration of the potential role of colchicine in regulating the growth and development of leguminous crops, including non-leguminous plant species [9,15,25].

Colchicine plays a vital role as a mutagenic agent, causing the multiplication of sets of plant chromosomes which has already become a method of choice for inducing genetic mutations (Figure 2). Plant polyploidisation gradually and naturally takes place as a method for speciation and formation of new species through the increase in the number of chromosomes [30]. The increase in the number of chromosomes using colchicine-induced polyploidisation was reported to result in plants showing enhanced sizes of morphological characters, such as larger leaves, flowers, and seeds (Table 1). Eng and Ho [3] also reported that this artificial polyploidisation can be used as a strategy for the adaptation of species, particularly of horticultural crops. In legumes, evaluation of nodule number, nodule size, terminal bacteroid differentiation, and symbionts quality (nodule environment, partner choice, host sanctions, etc.) revealed that plant polyploidy enhanced some key aspects of legume-rhizobia mutualism [31]. 

However, the use of this mechanistic hypothetical approach failed to explore the underlying mechanisms and influences of host benefits emanating from this mutualistic relationship of legumes with the involvement of rhizobia [31]. Nevertheless, this mutualism regulates the nutrient cycle in natural ecosystems and provides altered nitrogen (N) fixation rates, leading to further environmental adaptations. Although it is fully understood that plant polyploidy affects plant phenotype and genotype, much less is known about how it influences the interactions of plants with abiotic stresses, particularly, constraints such as drought. 

## 4. Growth Analysis of Drought-Stressed Polyploidised Leguminous Plants

As previously mentioned, the impact of drought stress on crop production continues to intensify, increasing yield losses and causing food production reductions and spikes in food prices. Many studies have reported that these reductions take place due to the detrimental effects that drought has on the growth and development of crop plants [22,32,33,34,35,36]. The cited reports discussed various ways in which drought impairs plant growth and development but also presented potential strategies in which this abiotic stress could be circumvented. To confer drought tolerance in legumes, crop plants can be improved through colchicine-induced polyploidisation (Figure 1 and Figure 2). As already discussed, this approach may lead to intraspecific variations in traits associated with drought stress. Colchicine causes changes in the morphological characteristics of roots and shoots and even in traits associated with plant responses to cope with progressive drought (Table 2). Earlier, Essel et al. [37] reported changes in quantitative characters, such as percentage germination, plant height, number of leaves, length of longest branches, and number of branches, including pod number and yield in cowpea plants developed from seeds treated with 0.05–0.20 g/dL of colchicine. 

Although colchicine had reduced the germination percentage in seeds treated with 0.10 g/dL, 0.15 g/dL, and 0.20 g/dL, improvements were reported in most of the quantitative characters of colchicine polyploidised cowpea plants. Similarly, colchicine-induced genetic variability in cowpea was observed through phenotypic changes in seedling emergence percentage, plant height, number of leaves, nodes, and survival percentage of the plantlets [38]. These morphological improvements have also been reported as valuable supplements to enhance plant responses to drought stress. For instance, the increase in plant height demonstrates increased plant cell growth that, if decreased, may alter water and nutrient potential and movement into or out of plant cells. As reported earlier by Nonami et al. [39], cell enlargement in combination with drought decreases this potential difference, subsequently inhibiting the growth of plants. Furthermore, water movement can be impeded by both water shortage and by the small cells observed during reduced plant growth. These abovementioned effects, like the number of leaves and branches, which were reported to signify improved plant response to drought stress have been considered. 

Plant leaves improve drought resistance by increasing wax coverage, cuticle thickness, and osmiophilicity [40] and reducing the number of branches, leading to combined effects of drought and exposure of plant shoots to photosynthetically active irradiation due to the overall reduction in plant architecture compared to water-stressed diploid plants. These observations were made in common bean (*Phaseolus vulgaris* L. cv. Berna) by Durigon et al. [41]. As such, drought remains one of the key factors restricting the successful establishment of effective plant architecture determined by the degree of branching, length of internodes, and shoot determinacy [42]. Therefore, plant polyploidisation can be thus used to improve plant shape or architecture, which is a primary determinant of growth, productivity, and yield of various leguminous crops. Additionally, traits such as leaf area, shoot and root biomass as well as the root length, as indicated in Table 2, also form part of the key plant adaptive strategy to enhance their carbon-sequestration capacity [43,44,45,46,47,48,49].

**Table 2:** Comparative evaluation of seed germination and morphology of a few polyploidised leguminous crops developed by imbibing seeds in colchicine solution for different durations, and responses from their diploid counterparts.

Parameter	Legumes	Colchicine (mg/L)	Duration (h)	Polyploids	Diploids	References
Germination (%)	Chickpea, cowpea, mung bean, soybean	0.025–0.5	3–6	90–92 *	90–100 *	Aliyu et al. [44]
Shoot height (cm)	Chickpea, cluster bean	0.1–15.0	4–24	8.02 *	4.42 *	Vijayalakshmi and Singh [45]
Branch no./plant	Cowpea, azuki bean	01–2.0	3–12	4.06	3.94	Ajayi et al. [46]
Leaf no./plant	Faba bean, winged bean, cluster bean	0.1–25.0	4–24	29.0 *	15.67 *	Ajayi et al. [46]
Leaf area (cm^2^)	Winged bean, faba bean	0.005–15	8–24	223.4 *	32.72 *	Udensi et al. [49]
Root length (cm)	Cluster bean	25.0	24	7.20 *	4.85 *	Vijayalakshmi and Singh [45]
Shoot biomass (g)	Soybean	0.15	2	3.15	2.96	Shehu et al. [10]
Root biomass (g)	Soybean	0.15	2	1.63 *	1.15 *	Shehu et al. [10], Essel et al. [37]

Note: Values within rows accompanied by asterisk are statistically significant at 0.05 *p*-value. Values are means calculated from the averages obtained from each parameter for each crop species.

## 5. Yield Evaluations in Polyploidised Legume Plants Affected by Drought Stress

As reported by a significant number of researchers, polyploidisation leads to improved yield components. In alfalfa (*Medicago sativa*), dry mass yields differed according to the cultivar used, and early harvest was also decreased due to water shortage [33]. It is well known that leguminous plants, regardless of their use as forage or grain crops, differ in terms of yield and other morpho-physiological parameters and remain highly drought-stress-sensitive (Table 3). As a result, drought stress has variable effects on the growth, productivity, and yield of these crops. Under water-stress-free conditions, polyploidised cowpea plants showed early flowering and fruiting, possibly due to the physiological changes caused by the mutagen, as reported by Essel et al. [37]. But the use of an increased colchicine concentration also interfered with maturity and early flowering. As expected, the efficiency of the plant polyploidisation induction system also depends on the colchicine concentration used, treatment duration, and the nature of the treated plant materials [30]. Other yield traits that were significantly influenced by the chemical mutagens applied included enhanced seed formation ability, number of pods per plant, and yield per plant, which were increased in some of the colchicine treatments compared to the control diploid plants [11,38].

Even though colchicine has been used to improve the growth of cowpea, mung bean, alfalfa, and other leguminous crops, as well as their various yield components, this mutation-inducing technique still needs to be thoroughly investigated for obtaining new useful traits and creating new genetic variability against abiotic stress. This genetic variability is what is needed in order to develop drought-resilient crops and achieve food security. However, in contrast, Vijayalakshmi and Singh [45] reported earlier the contrasting results of colchicine-induced reductions in fruiting and pod characters such as pod circumference, pod length, and pod weight in cluster bean [*Cyamopsis tetragonoloba* (L.) Taub]. Cluster bean serves as one of the most important major self-pollinated pulse crops in India, but its yield parameters were significantly decreased with the increasing concentrations of colchicine that were used to soak the seeds before sowing. 

Although colchicine and other mutagenic chemicals are required for rapid increases in the rates of yields over a reasonable period, mutagenesis through this chemical also revealed both negative and positive effects on crop growth and yields [46]. With the increased pressure of climate change and the frequency of severe drought events, other mutagenic chemicals such as sodium azide and gamma rays could be explored to increase genetic variability in agronomic traits and yields [47,48]. The use of such chemicals and techniques in mutation breeding was explored for two cowpea varieties treated with different doses of gamma rays and sodium azide [47]. The study reported improvements in the yield traits, as shown in Table 3, including the number of seeds per pod and seed weight, which positively correlated with an improved yield as a result of these treatments. As the global attention on legumes is based on yield quantity and quality, plant polyploidy can be used to minimise the production constraints of legumes, even under drought conditions.

## 6. Effect of Polyploidisation on Seed and Nutritional Quality

The twin effects of drought and heat stress primarily serve as major constraints in crop production and agricultural sustainability. These stresses obstruct productivity at various vegetative and reproductive phases of crop growth [52], but the seed filling stage suffers substantial damage. Drought affects yield as discussed in the previous sections (Table 3), simultaneously causing reductions in grain quality by affecting seed size and weight [53]. Seed filling is negatively influenced by reducing the ability of seed sink strength by decreasing the number of endosperm cells and amyloplasts formed, as reported by Sehgal et al. [52]. Furthermore, drought stress induces tissue senescence and enhances the remobilisation of photoassimilates from seed filling to the various plant parts affected by stress [54]. As anticipated, the drought-induced reduction in assimilates supply to developing grains significantly influences seed size and weight. 

In lentil (*Lens culinaris* Medikus), drought also interfered with nutritional quality by decreasing protein content and zinc (Zn) and iron (Fe) concentrations in the seeds [55]. As Sehgal et al. [52] further indicated, drought impaired mineral uptake, damaged membranes, and reduced photosynthetic rates ad stomatal conductance. These, then, have negative impacts on the production and mobilisation of assimilate nutrients to the developing seeds in various leguminous and non-leguminous crop species. Inhibited endosperm cell division and reduced numbers of starch granules markedly influence grain carbohydrate composition. Further effects include small size of starch grains, lower amylase content, and reduced nutrient translocation, mineral and ion transport, such as iron, zinc, sulfur (S), magnesium (Mg), and phosphorus (P), that are also highly required for serious enzymes related activities, and synthesis of primary as well as secondary metabolites [56,57,58,59]. 

In general, drought reduces nutrient acquisition and the concentrations of major nutrient uptake through the roots, which are correlated with the decrease in photoassimilation and redistribution from the source to the sink, especially during the seed filling stages of crop growth. Although the impact of water deficit on the seed and nutritional quality of grains is well characterised in legumes and other crops, very scant information is available on the seed and nutritional quality responses of plant polyploidy under drought stress conditions.

## 7. Ploidy Stability under Drought Stress Conditions

**Figure 3:**
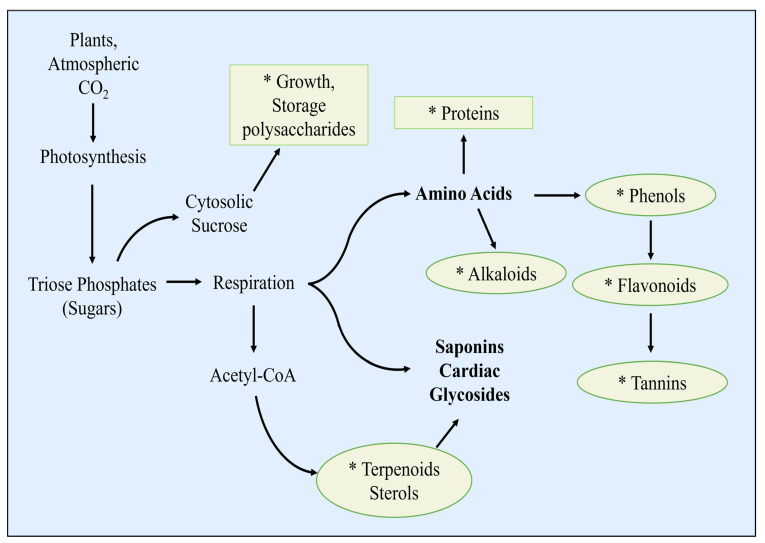
A schematic representation of metabolic synthesis involved in plant defence against drought stress. The pathways and secondary metabolites with asterisks demonstrate areas that are greatly influenced by the pattern of gene expression or duplication with or without drought stress in both diploid and polyploidised plants [66,67].

The hereditary control of plant growth is contained largely within the DNA of cell chromosomes. As the most powerful and versatile molecule, the DNA serves as a recipe coding for thousands of different proteins that help living organisms including plants to interact with each other and respond effectively to environmental stress challenges [60]. The DNA regulates, through the messenger RNA, the kinds of proteins and enzymes that function to control cell structure and plants’ responses to stress (Figure 3). Many previous and current studies have utilised DNA from different sources, often assembling it into gene constructs to drive biological research and biotechnological advances [61]. This includes tools such as exploring quantitative trait loci (QTL) and polyploidy induction for crop improvement. However, it is difficult to distinguish between the effects of natural speciation and those inflicted by artificial polyploidisation. Trait comparison, for instance, in characters that are the same in natural diploids and synthetic polyploids but different in naturally occurring polyploidised lineages can be helpful in identifying any existing negative effects in synthetic polyploids [62]. 

Colchicine, like trifluralin and oryzalin, operates by blocking the cell cycle in developing plant tissues; this can cause genome instability and many direct phenotypic consequences that are undesirable but can be eliminated through selective breeding [63]. Colchicine duplicates chromosome numbers, but it is still not clear how this influences the functions and maintenance of genes, including the expression of secondary metabolites (Figure 3). Plant secondary metabolites play a key role in crop responses to drought stress, but genome-wide studies of gene expression in polyploidised plants have demonstrated the differences in the composition and concentrations of metabolite profiles according to ploidy level. Although highly inconsistent, Gaynor et al. [64] found significant differences in plant secondary metabolites based on whole-genome duplication. Such effects on metabolism, as indicated in Figure 3, are responsible for the shifts in both physiological and phenotypic traits which confer tolerance to various ecological stress factors. As discussed in this paper, the improved morphological and physiological responses in plants as a result of increased genome size are hypothetically responsible for providing competitive advantages for growth and quick diversification in polyploids compared to their diploid counterparts [65]. 

Even though the routine use of artificial doubling of chromosome complements in legumes remains highly improbable, especially due to the recalcitrance in crops such as soybean, cowpea and faba bean, the effect of induced polyploidy in leguminous crops, as compared to other non-leguminous crop species, and the possible differential response of polyploidised varieties to environmental constraints will still present major challenges. This group of dicot crops overwhelmingly appear to fail in responding better to genetic improvement protocols, especially under the changing climatic conditions, because of the lack of routine and effective breeding systems [1,2,3,4,5,50,68]. While genetic engineering and induced polyploidisation continue to seem unachievable, stable and efficiently reproducible protocols for the development of legume polyploids remain a prerequisite to deal with the adverse effects of drought stress. Furthermore, polyploidy instability in these crops by far entails the general characterisation of leguminous polyploids that have failed to be established into the improved gene resources required for the further genetic manipulation of the individual crop species and cannot be tolerated. For instance, according to numerous cited reports, 58–100%, 4.42–8.02 cm, 1.43–44.5 g, and 7.61–4682.55 kg/ha of mean germination, plant height, 100-seed weight, and yield per hectare, compared to 90–100%, 4.85–7.20 cm, 0.39–38.5 g, and 6.85–2886 kg/ha of the same parameters were recorded in both polyploidised and diploid soybean plants, respectively [11,30,31,68]. The lack of trait uniformity in the polyploids, compared to diploid species, demonstrates the unequal and inconsistent distribution of chromosomes produced in reproductive cells containing varying degrees of chromosomal imbalances. Consequently, plants developed from these cells become almost completely sterile, with dysfunctional male and female reproductive cells that markedly indicate the instability of polyploid and aneuploidy leguminous plants.

## 8. Conclusions

The polyploidisation of leguminous crops using colchicine has been tested for almost more than a century across the globe. Colchicine remains the most preferred antimitotic agent in plant polyploidy production, compared to mutagenic chemicals such as sodium azide, oryzalin, and hydroxylamine. Although research on plant polyploidy has recently increased, information on the polyploidisation of leguminous crop plants remains elusive, particularly the responses of polyploids under drought stress conditions. This suggests that any new milestone achieved for both in vitro or in vivo or ex vitro colchicine-induced polyploidy will open new avenues for the genetic manipulation of these crops to confer stress tolerance. As previously reported, the current trend of polyploidisation in horticultural plants has already proceeded beyond addressing challenges involving the unequal distribution of chromosomes and studying the ploidy outcomes, as well as evaluating polyploidised plants through functional, metabolomic, and genomic studies. These approaches should also be encouraged and further explored for the genetic improvement of leguminous crops to confer tolerance to abiotic stress factors such as drought and salinity constraints in leguminous crops.

## Figures and Tables

**Table 3:** Estimates of yield components and biochemical parameters of diploid and polyploidised leguminous plants obtained by calculating averages for each parameter per plant.

Parameter	Polyploids	Diploids	Reference
No. of flowers	27.7	10.3	Udensi et al. [49]
No. of pods	33.0	19.33	Ikani et al. [50]
100-seed weight (g)	44.5	38.5	Aliyu et al. [44]
Yield/hectare (kg)	4682.6	2886.3	Esho et al. [51]
Proteins (µg/g)	223.4	32.72	Yadav et al. [11]
DNA (µg/g)	31.2	25.0	Yadav et al. [11]
RNA (µg/g)	245.0	190.0	Yadav et al. [11]

## Data Availability

Data that support the analysis and discussions made in this review paper are freely available as per citations and on various online research platforms.
